# Supplementary material for: vcferr: Development, validation, and application of a single nucleotide polymorphism genotyping error simulation framework
Source: F1000Res. 2022 Jul 11;11:775. [Version 1] doi: 10.12688/f1000research.122840.1 (PMC11109540; doi:10.12688/f1000research.122840.1)
Supplement: Peer review history

# Reviewer report: Reviewer response for version 1

**DOI:** 10.5256/f1000research.134883.r260018

**Published:** 2024-05-15

**Authors:** Cristiane Taniguti

**Affiliations:** 1 Texas A&M University, College Station, Texas, USA

The software presents a method for simulating genotyping errors in specific samples in a VCF file. The novelty compared to other software available is that it can introduce different error types and intensities. Having the tool in Conda or Docker is also a great advantage, as some other tools created for the same purpose are currently outdated and difficult to install.

 The authors provide two use cases, one measuring the impact of the different error types in kinship analysis and the other in GWAS and polygenic risk score estimation.

 The authors provide links for all mentioned resources including the software by itself, a companion package called nrc, and a Snakemake workflow to reproduce the analysis of the first use case.

 Results are presented for only the first of the cases. Despite not being necessary to understand the application, having access to results for the second use case would consolidate the statement.

 If not presently available, I recommend considering two additional features: incorporating a seed option for simulations and providing detailed information on the absolute number of modified genotypes and their final counts. Not knowing the absolute numbers, users can easily get lost in the interpretations of the results once the error rate parameters are based on frequencies and the absolute number will depend on the proportion of the initial genotypes, as emphasized in paragraph #12.

 I noticed that the introduction doesn't mention amplicon sequencing techniques like genotyping-by-sequencing (GBS or RAD-seq) as a means to obtain SNPs.  In the plant genetics field, GBS methods have been broadly used. Compared to the other methodologies mentioned in the text, GBS results have been showing a higher number of genotyping errors. Lots of efforts in terms of software have been made to try to overcome these errors (see Furuta et al. 2023 for an example Ref [1]). Briefly mentioning it in the text could expand the audience and the applications to this other domain.

 Other than predicting the accuracy rate for certain doubt quality samples, do you think that the tool could be useful to also measure the capabilities of these software designed to fix genotyping errors?

 Overall, the text is well-written and follows a logical description for a software release.

Are the conclusions about the tool and its performance adequately supported by the findings presented in the article?

Yes

Is the rationale for developing the new software tool clearly explained?

Yes

Is the description of the software tool technically sound?

Yes

Are sufficient details of the code, methods and analysis (if applicable) provided to allow replication of the software development and its use by others?

Yes

Is sufficient information provided to allow interpretation of the expected output datasets and any results generated using the tool?

Partly

Reviewer Expertise:

Bioinformatics, statistical genetics, plant genetics, polyploids

I confirm that I have read this submission and believe that I have an appropriate level of expertise to confirm that it is of an acceptable scientific standard, however I have significant reservations, as outlined above.

## References

[1] : GBScleanR: robust genotyping error correction using a hidden Markov model with error pattern recognition. *Genetics* .2023;224(2) : 10.1093/genetics/iyad055 10.1093/genetics/iyad055 36988327 PMC10213493

# Reviewer report: Reviewer response for version 1

**DOI:** 10.5256/f1000research.134883.r266345

**Published:** 2024-05-02

**Authors:** Gianluca Della Vedova

**Affiliations:** 1 University of Milano-Bicocca, Milan, Italy

The paper present a tool for simulating genotyping errors on top of a know vcf file. The tool has a clear scope and usefulness.

 The paper is well written and easy to follow. I have only a few, very minor, suggestions but I recommend to accept the paper.

 Page 3, "specified genotyping error". I suggest to state if you accept some kind of error profile.

 Page 3. "The tool is built using" -> "The tool depends on"

 Page 4. "from which error will be simulaed as the first positional argument". Please state explicitly that this is the ground truth you will use in your simulation steps.

 Page 4. (last paragraph) "The companion tool nrc ... registry". You have already discussed nrc and it docker image about half a page befor (just before Operation).

 Page 5, Code snippet #1. I expect a single "docker run" to suffice. May you explain why you also have a docker pull and a docker tag?

 Page 5, Code snippet #2. "Loop through ... error mode". I cannot find any loop in that code. Please make the code and the comments consistent.

 Page 7, Underlying data. Now that web browsers do not support ftp, I think it's time to move away from it, especially since it provides no advantages. That's not much that the authors can do, so it's mostly a suggestion for future works.

 Page 7. Please explain (here or somewhere else in the paper) why you have decided to release nrc as a docker image, instead of via conda (as you have done for vcferr). I find puzzling to use two different installation methods, so it's better to provide a rationale.

Are the conclusions about the tool and its performance adequately supported by the findings presented in the article?

Yes

Is the rationale for developing the new software tool clearly explained?

Yes

Is the description of the software tool technically sound?

Yes

Are sufficient details of the code, methods and analysis (if applicable) provided to allow replication of the software development and its use by others?

Yes

Is sufficient information provided to allow interpretation of the expected output datasets and any results generated using the tool?

Yes

Reviewer Expertise:

Algorithms

I confirm that I have read this submission and believe that I have an appropriate level of expertise to confirm that it is of an acceptable scientific standard.

# Reviewer report: Reviewer response for version 1

**DOI:** 10.5256/f1000research.134883.r235770

**Published:** 2024-02-20

**Authors:** Ardalan Naseri

**Affiliations:** 1 University of Texas Health Science Center at Houston, Texas, USA

The paper presents a new tool called vcferr, which can be used to simulate genotyping errors and missing data in variant call format (VCF) files. The authors demonstrate how vcferr can help answer research questions by introducing various levels and types of errors into a simulated pedigree, and evaluating how kinship analysis is affected. While there are other similar tools available, the authors highlight the unique features of their work.

 However, some minor clarifications are needed. The terms "degree" and "kind" of genotyping errors require further explanation.  I assume "Kind" refers to different error models, such as RARR or AARA, while "degree" refers to the rates. In the manuscript, they have been referred to as type as well.  A more coherent terminology would be helpful. 

 Regarding the implementation, "randomly draws from a list of possible genotypes (heterozygous, homozygous for the alternate allele, homozygous for the reference allele, missing) with each element weighted by error rates specified by the user.". It is unclear whether the values used for weighting are p_rarr, p_aar, etc., and if so, are the actual error rates produced by the tool the same? Would a p_rarr of 0.05 change exactly 5% of heterozygous sites to homozygous reference? 

 Additionally, it would be useful if the tool provided a seed value for reproducibility (if not available yet) when benchmarking other tools.

 Overall, the paper is well-written, and the tool provides a valuable resource for the research community.

Are the conclusions about the tool and its performance adequately supported by the findings presented in the article?

Yes

Is the rationale for developing the new software tool clearly explained?

Yes

Is the description of the software tool technically sound?

Yes

Are sufficient details of the code, methods and analysis (if applicable) provided to allow replication of the software development and its use by others?

Yes

Is sufficient information provided to allow interpretation of the expected output datasets and any results generated using the tool?

Yes

Reviewer Expertise:

Computational biology, Bioinformatics, Population genetics, Identity by descent, Artificial Intelligence

I confirm that I have read this submission and believe that I have an appropriate level of expertise to confirm that it is of an acceptable scientific standard, however I have significant reservations, as outlined above.
